# IFN-γR2 is strongly expressed on endothelial cells of gingival tissues from patients with chronic periodontitis

**DOI:** 10.1590/1678-7757-2017-0291

**Published:** 2018-10-02

**Authors:** Ramón Franco-Topete, José Sergio Zepeda-Nuño, Ana Lourdes Zamora-Perez, Martha Graciela Fuentes-Lerma, Belinda Claudia Gómez-Meda, Celia Guerrero-Velázquez

**Affiliations:** 1 Universidad de Guadalajara, Centro Universitario de Ciencias de la Salud, Departamento de Microbiología y Patología, Laboratorio de Patología, Guadalajara, México.; 2 Universidad de Guadalajara, Centro Universitario de Ciencias de la Salud, Departamento de Clínicas Odontológicas Integrales, Instituto de Investigación en Odontología, Guadalajara, México.; 3 Universidad de Guadalajara, Centro Universitario de los Altos, Departamento de Clínicas, Tepatitlán de Morelos, México.; 4 Universidad de Guadalajara, Centro Universitario de Ciencias de la Salud, Instituto de Biología Molecular en Medicina y Terapia Génica, Departamento de Biología Molecular y Genómica, Guadalajara, México.

**Keywords:** Interferon-gamma receptor 2, Interferon-gamma, Endothelial cells, Chronic periodontitis

## Abstract

Chronic periodontitis (CP) is characterized by gingival inflammation and bone destruction. It has been reported that interferon-gamma (IFN-γ) levels are high in CP patients; however, the IFN-γ receptor (IFN-γR) has not been studied in gingival tissue from these patients.

**Objective:**

To evaluate IFN-γ levels and IFN-γR expression in gingival tissue biopsies from chronic periodontitis patients compared with healthy subjects (HS).

**Material and Methods:**

Gingival tissues were obtained from all study subjects, CP (n = 18) and healthy subjects (HS) (n = 12). A tissue section of each study subject was embedded in paraffin blocks to determine the expression of IFN-γ R (IFN-γR1 and IFN-γR2) through immunohistochemistry. Another section of the tissue was homogenized and IFN-γ was measured by the ELISA technique.

**Results:**

No significant differences were found in the IFN-γR1 expression within the cell layers of the gingival tissue of the study groups. When analyzing the IFN-γR2 expression it was found that IFN-γR2 is strongly expressed in the endothelial cells of CP patients when compared to HS (p<0.05). IFN-γ concentrations in the gingival tissue were significantly higher in CP patients than in HS. No significant correlation between IFN-γ levels and the expression of IFN-γR1 and IFN-γR2 was found. However, a positive correlation between IFN-γ levels and clinical parameters [probing depth (PD) and clinical attachment level (CAL)] was found.

**Conclusion:**

The study of IFN-γR expression in gingival tissue samples from patients with CP showed an increase only in the IFN-γR2 chain in endothelial cells when compared to HS.

## Introduction

Chronic periodontitis (CP) is a strong immunoinflammatory response to periodontal pathogens influenced by metabolic disorders, ^1^ ^,^ ^2^ as well as genetic ^3^ ^,^ ^4^ and environmental factors. ^5^

Chronic gingival and periodontal inflammations lead to attachment loss with a periodontal pocket formation and progressive alveolar bone destruction. ^6^ ^,^ ^7^

Responding to the bacterial infection in periodontitis, antigen-presenting cells (APC) produce IL-12 and IL- 18. ^8^ Subsequently, IL-12 and IL-18 synergize and stimulate the production of interferon-gamma (IFN-γ) in T and NK cells. ^9^ ^-^ ^11^ IFN-γ can bind autocrinally to T/ NK cells or paracrinally to macrophages/APC through its specific receptor (IFN-γR), which consists of four chains: two alpha chains (IFN-γR1) responsible for capturing IFN-γ, and two beta chains (IFN-γR2) involved in the JAK-STAT signaling pathway. ^12^

IFN-γ and IFN-γR are known to be fundamental in the pathophysiology of several chronic and infectious diseases. In this sense, a deregulation in the expression of IFN-γ and IFN-γR levels in patients with lepromatous leprosy has been described. ^8^ ^,^ ^13^ In this regard, an increase in mRNA and IFN-γ protein has been reported in gingival tissue, gingival crevicular fluid (GCF), and serum samples from CP patients compared to healthy subjects (HS). ^14^ ^-^ ^19^ Regarding the expression of IFN-γR in CP, only studies of IFN- γR1 polymorphisms exist and no association has been found for periodontitis. ^20^ ^,^ ^21^ Therefore, the objective of this study was to evaluate IFN-γ levels and the expression of IFN-γR in gingival tissue samples from CP patients compared to healthy subjects.

## Material and methods

### Study subjects

Thirty subjects who attended the Periodontal Clinic of the Dentistry School were recruited to participate in this study. Patients who had clinical features typical of chronic periodontitis (as described later), were enrolled consecutively. All subjects were in good general health and had not received previous periodontal therapy or taken antibiotics, immunomodulatory, or anti-inflammatory drugs in the six months prior to the study. Pregnant women, smokers, patients who were undergoing antibiotic prophylaxis for dental treatment, patients with any systemic disease, or those who were on long-term medication that could affect the expression of gingivitis or periodontitis were excluded from the study. ^11^ ^,^ ^22^

The purpose of this study was explained to each subject before he/she agreed to participate, and their informed consent was obtained according to the World Medical Association Declaration of Helsinki v. 2002. The study was approved by the Medical Ethical Review Committee and all subjects gave their written approval before participating based on the General Health Law. ^11^

### Clinical examination

Clinical examinations were performed on all existing teeth of the participants and periodontal conditions were assessed based on the following parameters: sites with plaque (SP), bleeding on probing (BOP), probing depth (PD) and clinical attachment level (CAL). The examination of all subjects was performed using a periodontal probe (15 mm, probe tip diameter 0.5 mm; University of North Carolina UNC-15 Hu-Friedy^®^, Chicago, IL, USA) (Hu-Friedy, Chicago, IL, USA) by a single researcher, and the mean sulcular depth was calculated. ^3^ ^,^ ^11^

Clinical parameters were obtained on six sites *per* tooth, and the results were expressed as percentage of sites (for plaque and BOP) and as mean ± standard deviation (SD) for PD and CAL. ^11^ ^,^ ^22^ ^,^ ^23^ Periapical radiographs were performed by the same researcher using the long-cone paralleling technique with the same radiographic equipment, film, exposure and development conditions for all subjects. ^2^

### Study groups

Medical and dental records were obtained from participants and diagnosis was performed according to the classification of the American Academy of Periodontology. ^11^ ^,^ ^22^ ^,^ ^23^

Healthy subjects group. This group consisted of 12 HS (eight women and four men; 35.2±4.5 mean age) who attended the clinic for esthetic surgery and had no evidence of CAL, clinical inflammation, BOP, or radiographic evidence of bone loss. The gingival tissue of this group was defined as clinically healthy when the mean sulcular depth was ≤3 mm and there was no evidence of BOP on any surface. ^11^ ^,^ ^23^

Chronic periodontitis group. This group consisted of 18 patients (ten women and eight men; 44.2±7.3 mean age) who presented localized pockets at various sites in the oral cavity, including ≥5 mm CAL and ≥6 mm PD. ^11^ ^,^ ^22^ ^,^ ^23^

### Sample collection and surgical procedures

Collection of gingival tissue from HS was performed for esthetic indications (crown lengthening), and from CP patients during the surgical phase. ^24^ The gingival tissues were collected by routine periodontal surgery, as described below: two periodontist surgeons performed all operations. All patients were prepared with chlorhexidine gluconate for two minutes before the surgical procedure. ^6^ The surgical procedure was performed using a local anesthetic (2% articaine with 1:100,000 epinephrine). ^6^ For CP patients, sulcular incisions were made on all teeth and mucoperiosteal flaps were designed in the lingual and palatal areas using a prichard curette (Hu-Friedy^®^, Chicago, IL, USA) and a periostal elevator (9 Molt Periosteal Elevator Black Line, Hu-Friedy^®^, Chicago, IL, USA). ^6^ The flaps were designed on all affected teeth of the quadrant, preserving the connective gingival tissue as much as possible. Once the flap was reflected, the gingival sulcus, junctional epithelium and adjacent connective tissue were carefully removed using LaGrange scissors (Hu-Friedy^®^, Chicago, IL, USA), tissue scissors and a Lucas curette (Hu-Friedy^®^, Chicago, IL, USA). Hand, ultrasonic and diamond rotating instruments, and finishing burs were used for the debridement of soft tissues and brushing of roots. ^6^ For HS, submarginal and sulcular incisions were performed with a size 15 Bard-Parker blade, then, the gingival tissue was carefully removed with a Lucas curette (Hu-Friedy^®^, Chicago, IL, USA), the flap was reflected, and the osteotomy and osteoplasty were performed according to the needs of each healthy subject.

Flap edges were approximated and sutured with 6-0 Vicryl thread and subsequently, pressure was applied in the area for three minutes. Patients were prescribed amoxicillin 1000 mg/day for 6 days, as well as an oral rinse with 0.20% chlorhexidine gluconate twice a day for two weeks. ^6^ The sutures were removed 10 days after the surgery if edge interface stability of the flap was verified. Plaque control and supragingival cleansing were performed weekly over the first six weeks after surgery. Finally, patients were called at six and twelve months to reinforce care-hygiene measures.

A section of each tissue sample was placed in a 1.5 mL microtube. The microtubes were immersed in crushed ice (4°C) and immediately transported to the laboratory, being stored in an ultrafreezer at −80°C until analysis. The other section of the tissue was placed in a paraformaldehyde buffer and sent to a histopathologist for the embedding process in paraffin blocks.

### IFN-γR expression (immunohistochemistry)

The detection of IFN-γR1 and IFNγR2 was performed using a direct immunohistochemical method. Serial sections (6 mm×5 mm) from the formalin-fixed, paraffin-embedded blocks were used. Thin sections (5 μm) were deparaffinized in xylene, rehydrated through a graded series of ethanol and heated in citrate buffer (10 mM; pH 6.0) in a steamer for 30 minutes for antigen retrieval. ^24^

The slides were washed in phosphate buffered saline (PBS), treated with peroxidase quenching solution (Life Technologies, Camarillo, CA, USA) for five minutes at room temperature and washed with tris-buffered saline (TBS) for five minutes. The sections were incubated with anti-IFN-γR1 or anti-IFN-γR2 (Santa Cruz Biotechnology, Santa Cruz, CA, USA) antibodies diluted 1:50 and 1:200, respectively, in TBS for 90 minutes at room temperature. This assay was performed in triplicate. For immunohistochemical staining, the Histostain^®^-Plus 3rd Gen IHC Detection Kit (Life Technologies, Camarillo, CA, USA) was used according to the manufacturer's instructions. After incubation with the primary antibody, each sample was incubated for 10 min with a biotinylated secondary antibody, followed by incubation with a streptavidin-peroxidase conjugate for 10 minutes. The DAB chromogen was used as the substrate. All sections were counterstained with hematoxylin QS (Vector Laboratories Inc, Burlingame, CA, USA), and examined under light microscopy with a grid eyepiece. Negative control sections were treated similarly but without primary antibodies. ^24^

### Evaluation of immunohistochemical staining

Histological and immunohistochemical evaluations were performed independently by two pathologists. The staining reaction was evaluated using the immunoreactive score (IRS). ^24^ ^,^ ^25^ IRS=SI (staining intensity)×PP (percentage of positive cells). SI was determined as 0 (negative), 1 (weak), 2 (moderate) and 3 (strong). PP was defined as 0 (negative), 1 (1-10% positive cells), 2 (11-50% positive cells), 3 (51-75% positive cells) and 4 (76 – 100% positive cells). ^24^

### IFN-γ levels (enzyme-linked immunosorbent assay)

#### Tissue preparation for protein extraction

The frozen gingival tissues were solubilized. Briefly, the tissue was blotted, weighed on a microbalance, cut into small pieces (1-2 mm^3^) with a scalpel and then, placed in a 1.5 mL microtube with enough PBS volume to ensure the following dilution: 10 mg tissue /100 ml PBS plus protease inhibitor (Sigma Chemicals, St Louis, MO, USA). Subsequently, the tissues were macerated with a polypropylene pestle and a vortex. Following, the sample was centrifuged at 600 *g,* and the supernatant was frozen at −70°C until the analysis by a cytokine ELISA kit. The procedure was performed at 4°C. ^18^ ^,^ ^24^

#### Protein assay

A standard Bradford micromethod was used to assess the protein concentration in each gingival sample. ^11^ ^,^ ^26^ Absorbance was read at 570 nm in a microplate spectrophotometer. Protein concentrations were calculated from a bovine serum albumin standard curve (Sigma Chemical, St Louis, MO, USA) and expressed as mg/mL. The Bradford reagent used consisted of 100 mg of Coomassie brilliant blue G-250 (Research Organics, Cleveland, OH, USA), 50 mL of 95% ethanol (Caledon Laboratories Ltd, Georgetown, ON, Canada), 100 mL of concentrated phosphoric acid (Caledon Laboratories Ltd Georgetown, ON, Canada) and 200 mL of distilled water. ^11^

To measure IFN-γ levels, aliquots from either tissue homogenates or cytokine standards were added in triplicate to the wells of microtiter plates to determine the concentrations of human IFN-γ (Peprotech, Rocky Hill, NJ, USA). ^11^ The absorbance of each well was read at 405 nm in a microplate spectrophotometer, and the IFN-γ concentration was calculated from the standard curve included in each assay kit. Antiserum-specificity controls were included in each assay. Controls for the plate-to-plate variation were also used when appropriate. The concentrations of human IFN-γ were expressed as pg/mg of gingival tissue. ^11^

### Statistical analysis

The results are expressed as mean±standard deviation for age, PD, CAL, IFN-γ levels and IFN- γR1/R2 expression. The data presented an abnormal distribution using the Shapiro-Wilk test for a small sample size and the analysis was using the nonparametric Mann-Whitney *U* -test. The results for gender, SP and BOP are presented as percentage; a Chi-square test was used to compare this data in CP *vs* HS groups. Spearman's correlation was used to study the relation between IFN-γ levels, IFN-γR1/ R2 expression and clinical findings. ^11^ All results were analyzed using the SPSS 20.0v software (SPSS. Chicago, IL, USA). Significance was considered when *P* <0.05.

## Results

Table 1 shows demographic and clinical data of the participants. The mean (in years) was statistically significantly higher in the CP group (44.2±7.3) than in the HS group (35.2±4.5). CP patients showed higher percentages of SP and BOP, while in the HS these were not observed. PD and CAL were significantly higher in CP patients when compared to HS ( Table 1 ).

**Table 1:** Mean±S.D. and percentages of demographic and clinical data from patients with chronic periodontitis (CP) and healthy subject (HS)

Parameters/Group	HS (n=12)	CP (n=18)	P-value *
Mean age (years)	35.2±4.5	44.2±7.3	<0.01 *
Male/Female	4/8	8/10	
SP (%)	0	100	<0.001 *
BOP (%)	0	100	<0.001 *
PD (mm)	1.6±0.5	5.4±1.0	<0.01 *
CAL (mm)	1.1±0.3	8.8±0.8	<0.01 *

* Significance level P<0.05

Chi-square test was used to compare BOP and CAL Mann-Whitney U-test was used to compare age, PD and CAL BOP=bleeding on probing; CAL=clinical attachment level; CP=chronic periodontitis; HS=healthy subjects; NS=non-significant; PD=probing depth; SD=standard deviation; SP=sites with plaque

**Figure 1:**
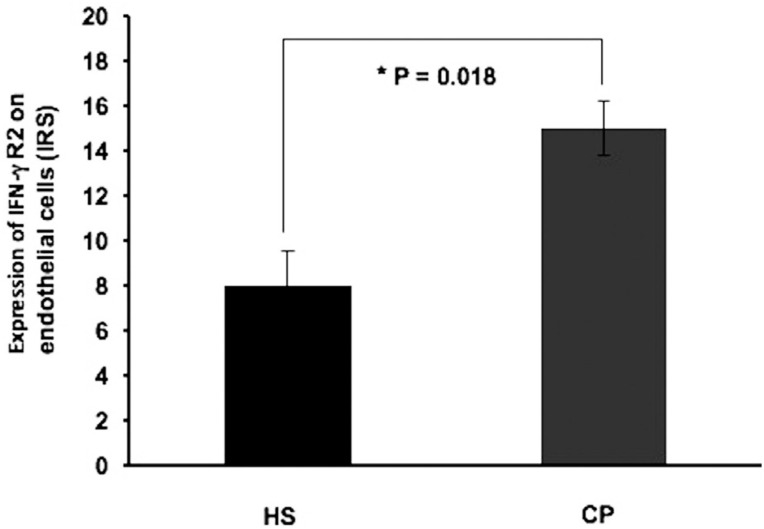
IFN-γR2 expression on endothelial cells from study subjects. The results are presented as mean±SD. We found a significantly high IFN-γR2 expression in endothelial cells of gingival tissue from patients with chronic periodontitis when compared to healthy subjects CP: Chronic periodontitis HS: Healthy subjects IRS: Immunoreactive score *Significance level (P<0.05)

**Figure 2:**
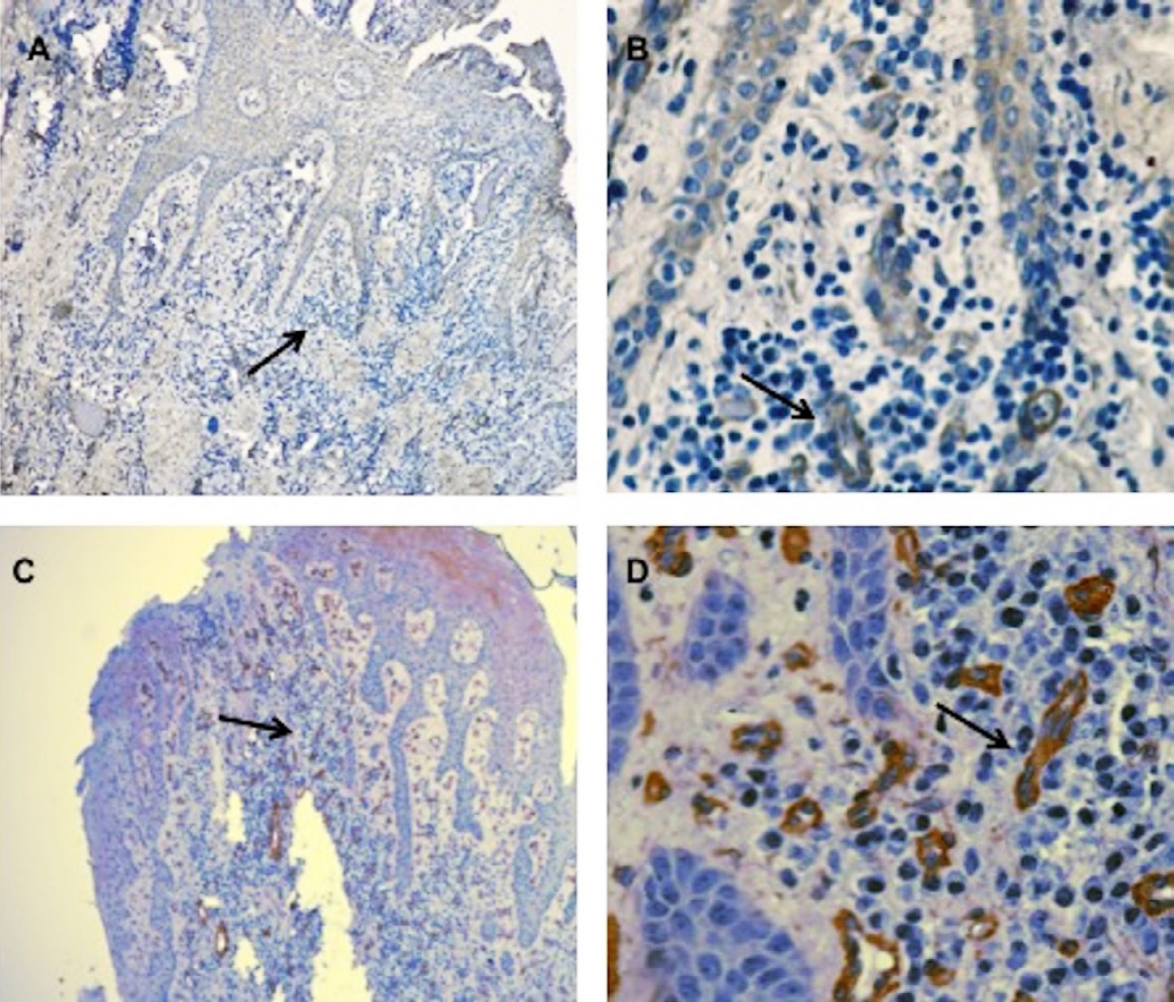
Cellular localization of IFN-γR2 on gingival tissues in chronic periodontitis patients (CP) and healthy subjects (HS) by immunohistochemistry. Surface expression of IFN-γR2 in endothelial cells of healthy subjects (A, B); and surface expression of IFN-γR2 in endothelial cells of CP patients (C, D); we must highlight that IFN-γR2 is strongly expressed in the endothelial cells of CP patients (B) when compared to HS (D), as well as an increase in the vasculature in the gingival tissue of CP patients (C and D) when compared to HS (A and B). (A and C, original magnification x10); (B and D, original magnification x60). Hematoxylin counterstained

IFN-γR1/R2 expression results for immunohistochemistry are presented as the mean±SD of IRS of the study groups. No statistically significant differences were observed in IFN-γR1 expression in the cell layers of the gingival tissue of the study groups ( Table 2 ). Regarding IFN-γR2 expression, no statistically significant differences were found in surface epithelium (4.1±1.2), intermediate epithelium (5.9±3.7) and basal epithelium (2.6±1.8) in CP patients, the values for HS were: surface epithelium (3.6±2.4), intermediate epithelium (7.2±5.6) and basal epithelium (1.8±1.2). No significant differences were found in the IFN-γR2 expression in mononuclear cells between CP patients (1.2±0.8) and HS (1.3±0.9). In contrast, IFN-γR2 was found to be strongly expressed in the endothelial cells of CP patients (15±1.2) when compared to HS (8±1.5) ( *P* =0.018) ( Table 3 ) ( Figure 1 and Figure 2 ).

**Table 2:** IFN-γR1 expression in epithelium and stroma of healthy subjects (HS) and patients with chronic periodontitis (CP) (Mean±SD)

IFN-γR1 expression	HS (n=12)	CP (n=18)	P value
IE (IRS)	6.6±1.8	4.3±3.0	NS
BE (IRS)	1.6±0.5	1.0±0.9	NS
MNC (IRS)	0.8±0.4	1.0±1.0	NS
EC (IRS)	1.9±0.9	2.4±2.0	NS
F (IRS)	2.5±1.4	2.4±2.0	NS

* Significance level P<0.05

Mann-Whitney U-test was used to compare IFN-γR1 expression BE=basal epithelium; CP=chronic periodontitis; EC=endothelial cells; F=fibroblast; HS=healthy subjects; IE=Intermediate epithelium; IRS=immunoreactive score; MNC=mononuclear cells; NS=non-significant; SD=standard deviation

**Table 3:** IFN-γR2 expression in epithelium and stroma of healthy subjects (HS) and patients with chronic periodontitis (CP) (Mean±SD)

IFN-γR2 expression	HS (n=12)	CP (n=18)	P value
SE (IRS)	3.6±2.4	4.1±1.2	NS
IE (IRS)	7.2±5.6	5.9±3.7	NS
BE (IRS)	1.8±1.2	2.6±1.8	NS
MNC (IRS)	1.3±0.9	1.2±0.8	NS
EC (IRS)	8.0±1.5	15.0±1.2	* P=0.018

* Significance level P<0.05

BE=basal epithelium; CP=chronic periodontitis; EC=endothelial cells; HS=healthy subjects; IE=Intermediate epithelium; IRS=immunoreactive score; MNC=mononuclear cells; NS=non-significant; SD=standard deviation; SE=superficial epithelium

**Figure 3:**
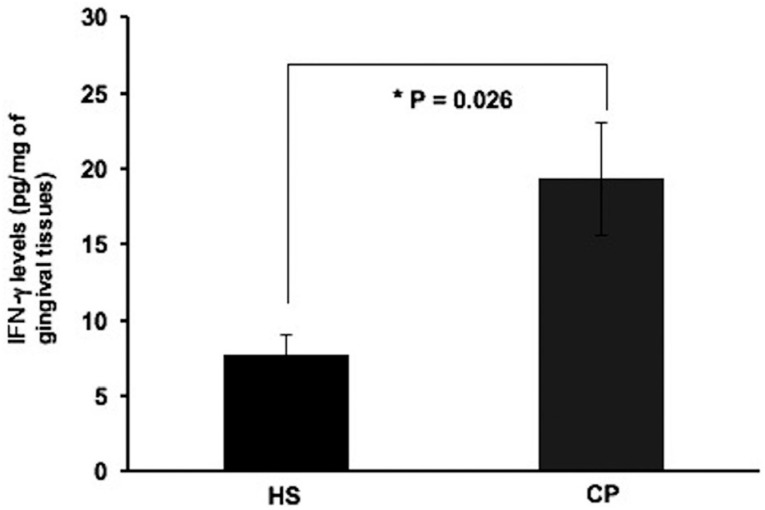
IFN-γ levels in healthy subjects (HS) and chronic periodontitis (CP). The IFN-γ levels were high in CP patients when compared to HS. IFN-γ levels were detected using ELISA in gingival tissue (GT) biopsies. The results are shown as mean±SD * Significance level (P<0.05)

IFN-γ levels in gingival tissues were higher in CP patients (19.3±3.7 pg/mg of gingival tissue) than in HS (7.6±1.3 pg/mg of gingival tissue) ( *P* =0.026) ( Figure 3 ).

Correlation analysis only showed a significantly positive correlation between IFN-γ levels with PD [(r=0.62) (P=0.048)] and CAL [(r=0.58) (P=0.045)] in CP patients.

## Discussion

IFN-γR1 and IFN-γR2 are expressed in several cells, including human endothelial cells. ^27^ ^,^ ^28^ Although little is known about the intracellular trafficking of IFN-γR2, it seems that on certain cell types, IFN-γ regulates the expression of IFN-γR2 and also determines the ability of these cells to respond to subsequent exposure to IFN-γ. ^12^ ^,^ ^29^

In this study, no statistically differences were found for IFN-γR1 expression in the gingival tissue of CP patients and HS. This result corroborates with the literature, since IFN-γR1 is constitutively expressed in several cells, even in some that do not have a primary immune function. ^12^ ^,^ ^29^

On the other hand, high levels of mRNA and IFN-γ have been found in different samples (gingival tissue, serum, saliva, and GCF) of CP patients when compared to HS. ^14^ ^-^ ^19^ This study found high levels of IFN-γ in the gingival tissues of CP patients when compared to HS, corroborating these studies. We must highlight that only Górska, et al. ^17^ (2003) and Johnson, et al. ^18^ (2005) have detected elevated IFN-γ levels in gingival tissue samples from CP patients. These authors reported the IFN-γ levels in pg/mL; in this study we decided to report IFN-γ in pg/mg of gingival tissues by ELISA. Similarly, IFN-γ concentrations were found to be significantly higher in CP patients than in HS. Given this context, it has been described that high IFN-γ levels in the infected periodontium are associated with the progression of lesions and the severity of inflammatory diseases. ^21^ In addition, the antigen stimuli and pathways lead to chronic elevation of IFN-γ and maintain the activation of resident phagocytes in the periodontium through the secretion of metalloproteinases (MMP) and other molecules, which leads to increased bone destruction. ^16^ In this sense, we found a significant correlation between high IFN-γ levels in gingival tissue and clinical parameters (PD and CAL) in the CP group, confirming that elevated IFN-γ levels in CP patients are related with bone destruction in the periodontium.

Interestingly, we found that IFN-γR2 was expressed in the endothelial cells of gingival tissue of both groups, corroborating a study that describes high IFN-γR2 expression in aortic endothelial cells. ^27^ ^,^ ^28^ However, the staining intensity of IFN-γR2 was significantly stronger in CP patients than in HS. To date, there are no studies explaining the high IFN-γR2 expression in endothelial cells in chronic periodontitis. However, we know that IFN-γ regulates the expression of IFN- γR2. ^12^ ^,^ ^29^ In this regard, we can assume that elevated IFN-γ levels have influence in the over expression of IFN-γ R2 in endothelial cells in gingival tissue samples from CP patients. ^7^ ^,^ ^14^ ^,^ ^15^ ^,^ ^18^ ^,^ ^20^ ^,^ ^28^ However, we found no significant correlation between IFN-γ levels and IFN- γR2 expression in the endothelial cells of CP patients.

It is known that in engineered endothelial cells, IFN-γ binding to IFN-γR1 assembles IFN-γR2 and activates JAK-STAT signaling to promote vascular growth and increase. ^27^ In this sense, CP patients present an increase in angiogenesis, the strong expression of IFN-γR2 in endothelial cells is probably linked to such increase, as well as the elevated IFN-γ levels in chronic periodontitis. ^18^ ^,^ ^27^ ^,^ ^30^

Finally, further studies on molecular mechanisms are required to determine the role of over expression of IFN-γ and IFN-γR2 in endothelial cells of gingival tissue from CP patients and the relation with angiogenesis and tissue destruction in periodontal disease.

## Conclusions

Strong IFN-γR2 expression was observed in endothelial cells, as well as elevated IFN-γ levels in gingival tissues biopsies from CP patients when compared to HS. We found a positive correlation between IFN-γ and clinical parameters in CP.

## References

[1] Lucarini G, Tirabassi G, Zizzi A, Balercia G, Quaranta A, Rubini C (2016). Uncoupling of vascular endothelial growth factor (VEGF) and inducible nitric oxide synthase (iNOS) in gingival tissue of type 2 diabetic patients. Inflammation.

[2] Zizzi A, Tirabassi G, Aspriello SD, Piemontese M, Rubini C, Lucarini G (2013). Gingival advanced glycation end-products in diabetes mellitus-associated chronic periodontitis: an immunohistochemical study. J Periodontal Res.

[3] Aspriello SD, Zizzi A, Tirabassi G, Buldreghini E, Biscotti T, Faloia E (2011). Diabetes mellitus-associated periodontitis: differences between type 1 and type 2 diabetes mellitus. J Periodontal Res.

[4] Lucarini G, Zizzi A, Ferrante L, D'Angelo AB, Rubini C, Aspriello SD (2015). CD133 expression could be a predictive marker of periodontal regeneration. J Biol Regul Homeost Agents.

[5] Zizzi A, Piemontese M, Gesuita R, Nori A, Berlin RS, Rocchetti R (2014). Periodontal status in the Down's syndrome subjects living in central-eastern Italy: the effects of place of living. Int J Dent Hyg.

[6] Aspriello SD, Ferrante L, Rubini C, Piemontese M (2011). Comparative study of DFDBA in combination with enamel matrix derivative versus DFDBA alone for treatment of periodontal intrabony defects at 12 months post-surgery. Clin Oral Investig.

[7] Piemontese M, Aspriello SD, Rubini C, Ferrante L, Procaccini M (2008). Treatment of periodontal intrabony defects with demineralized freeze-dried bone allograft in combination with platelet-rich plasma: a comparative clinical trial. J Periodontol.

[8] Guerrero-Velázquez C, Lopez-Roa RI, Delgado-Rizo V, Guillen-Vargas CM, Montoya-Buelna M, Fafutis-Morris M (2010). Abnormalities in intracellular processing and expression of interferon-gamma receptor in adherent cells from lepromatous leprosy patients. J Interferon Cytokine Res.

[9] Nakahira M, Ahn HJ, Park WR, Gao P, Tomura M, Park CS (2002). Synergy of IL-12 and IL-18 for INF-gamma gene expression: IL-12-induced STAT4 contributes to INF-gamma promoter activation by up-regulating the binding activity of IL-18-induced activator protein 1. J Immunol.

[10] Okamura H, Tsutsui H, Kashiwamura S, Yoshimoto T, Nakanishi K (1998). Interleukin-18: a novel cytokine that augments both innate and acquired immunity. Adv Immunol.

[11] Sánchez-Hernández PE, Zamora-Perez AL, Fuentes-Lerma M, Robles-Gómez C, Mariaud-Schmidt RP, Guerrero-Velázquez C (2011). IL-12 and IL-18 levels in serum and gingival tissue in aggressive and chronic periodontitis. Oral Dis.

[12] Bach EA, Aguet M, Schreiber RD (1997). The IFN gamma receptor: a paradigm for cytokine receptor signaling. Annu Rev Immunol.

[13] Lopez Roa RI, Guerrero Velásquez C, Alvarado Navarro A, Montoya Buelna M, Garcia Niebla C, Fafutis Morris (2008). Recovery of IFN-gamma levels in PBMCs from lepromatous leprosy patients through the synergistic actions of the cytokines IL-12 and IL-18. Int Immunopharmacol.

[14] Cavalla F, Biguetti C, Colavite PM, Silveira EV, Martins W, Letra A (2015). TBX21-1993T/C (rs4794067) polymorphism is associated with increased risk of chronic periodontitis and increased T-bet expression in periodontal lesions, but does not significantly impact the IFN-γ transcriptional level or the pattern of periodontophatic bacterial infection. Virulence.

[15] Cavalla F, Biguetti CC, Dionisio TJ, Azevedo MC, Martins W, Santos CF (2017). CCR5Δ32 (rs333) polymorphism is associated with decreased risk of chronic and aggressive periodontitis: a case-control analysis based in disease resistance and susceptibility phenotypes. Cytokine.

[16] Dutzan N, Vernal R, Hernandez M, Dezerega A, Rivera O, Silva N (2009). Levels of interferon-gamma and transcription factor T-bet in progressive periodontal lesions in patients with chronic periodontitis. J Periodontol.

[17] Górska R, Gregorek H, Kowalski J, Laskus-Perendyk A, Syczewska M, Madaliński K (2003). Relationship between clinical parameters and cytokine profiles in inflamed gingival tissue and serum samples from patients with chronic periodontitis. J Clin Periodontol.

[18] Johnson RB, Serio F (2005). Interleukin-18 concentrations and the pathogenesis of periodontal disease. J Periodontol.

[19] Souto GR, Queiroz-Junior CM, Abreu MH, Costa FP, Mesquita RA (2014). Pro-inflammatory, Th1, Th2, Th17 Cytokines and dendritic cells: a cross-sectional study in chronic periodontitis. PLos One.

[20] Fraser DA, Loos BG, Boman U, van Winkelhoff AJ, van der Velden U, Schenck K (2003). Polymorphisms in an interferon-gamma receptor-1 gene marker and suceptibility to periodontitis. Acta Odontol Scand.

[21] Heidari Z, Mahmoudzadeh-Sagheb H, Hashemi M, Ansarimoghaddam S, Moudi B, Sheibak N (2015). Association between IFN-γ +874A/T and IFN- γR1 (-611A/G, +189T/G, and +95C/T) gene polymorphisms and chronic periodontitis in a sample of Iranian population. Int J Dent.

[22] Löe H (1967). The gingival index, the plaque index and the retention index systems. J Periodontol.

[23] Armitage GC (1999). Development of a classification system for periodontal diseases and conditions. Ann Periodontol.

[24] Sánchez-Hernández PE, Ramirez-Dueñas MG, Albarran-Somoza B, García-Iglesias T, del Toro-Arreola A, Franco-Topete R (2008). Protease-activated receptor-2 (PAR-2) in cervical cancer proliferation. Gynecol Oncol.

[25] Ikeda O, Egami H, Ishiko T, Ishikawa S, Kamohara H, Hidaka H (2003). Expression of proteinase-activated receptor-2 in human pancreatic cancer: a possible relation to cancer invasion and induction of fibrosis. Int J Oncol.

[26] Bradford MM (1976). A rapid and sensitive method for the quantitation of microgram quantities of protein utilizing the principle of protein-dye binding. Anal Biochem.

[27] Methe H, Edelman ER (2006). Tissue engineering of endothelial cells and the immune response. Transplant Proc.

[28] Valente G, Ozmen L, Novelli F, Geuna M, Palestro G, Forni G (1992). Distribution of interferon-gamma receptor in human tissues. Eur J Immunol.

[29] Sakatsume M, Finbloom DS (1996). Modulation of the expression of the IFN-gamma receptor beta-chain controls responsiveness to IFN-gamma in human peripheral blood T cells. J Immunol.

[30] Artese L, Piattelli A, Gouveia Cardoso LA, Ferrari DS, Onuma T, Piccirilli M (2010). Immunoexpression of angiogenesis, nitric oxide synthase, and proliferation markers in gingival samples of patients with aggressive and chronic periodontitis. J Periodontol.

